# Methane Production and Methanogenic Archaea in the Digestive Tracts of Millipedes (Diplopoda)

**DOI:** 10.1371/journal.pone.0102659

**Published:** 2014-07-16

**Authors:** Vladimír Šustr, Alica Chroňáková, Stanislava Semanová, Karel Tajovský, Miloslav Šimek

**Affiliations:** 1 Institute of Soil Biology, Biology Centre AS CR, v.v.i., České Budějovice, Czech Republic; 2 Faculty of Science, University of South Bohemia, České Budějovice, Czech Republic; Universidade Federal do Rio de Janeiro, Brazil

## Abstract

Methane production by intestinal methanogenic Archaea and their community structure were compared among phylogenetic lineages of millipedes. Tropical and temperate millipedes of 35 species and 17 families were investigated. Species that emitted methane were mostly in the juliform orders Julida, Spirobolida, and Spirostreptida. The irregular phylogenetic distribution of methane production correlated with the presence of the methanogen-specific *mcrA* gene. The study brings the first detailed survey of methanogens’ diversity in the digestive tract of millipedes. Sequences related to Methanosarcinales, Methanobacteriales, Methanomicrobiales and some unclassified Archaea were detected using molecular profiling (DGGE). The differences in substrate preferences of the main lineages of methanogenic Archaea found in different millipede orders indicate that the composition of methanogen communities may reflect the differences in available substrates for methanogenesis or the presence of symbiotic protozoa in the digestive tract. We conclude that differences in methane production in the millipede gut reflect differences in the activity and proliferation of intestinal methanogens rather than an absolute inability of some millipede taxa to host methanogens. This inference was supported by the general presence of methanogenic activity in millipede faecal pellets and the presence of the 16S rRNA gene of methanogens in all tested taxa in the two main groups of millipedes, the Helminthophora and the Pentazonia.

## Introduction

About 600 Tg of the greenhouse gas methane is produced on Earth each year [1]. Although methane, which has the second-largest impact on global warming after CO_2_ [2], is released to the atmosphere from human activities [3] including industry, agriculture, and waste management [4], natural sources account for 37% of global methane emissions. All methane, other than that from industry, is produced by the only known biogenic source - methanogenic Archaea. Methanogenic Archaea are an ancient group of microorganisms that occupy ecological niches with limited oxygen concentrations such as wetlands, rice fields, swamps, marshes, freshwater and marine sediments, agricultural soils, and the digestive tracts of humans and other animals [5], [6], [7], [8], [9].

Many authors have estimated methane production from vertebrates [10], [11], [12], [13], and other authors have discussed the global importance of methane originating from invertebrate hosts and its potential contribution to the increase in atmospheric methane [14], [15], [16], [17]. Termites have been recognized as a globally important source of methane in that they are estimated to contribute between 5 and 19% of the global methane emissions [18]. Methane production and the presence of methanogens has also been systematically screened in a wide variety of invertebrate taxa [19], [20], [21], [22], [23]; the results indicate that symbiotic associations involving methanogens and methane production are likely to be a characteristic property of the host taxon and that methane production is restricted to millipedes, cockroaches, beetles (Cetonidae), and termites [19].

Using microscopy and specific autofluorescence, Hackstein and Stumm [19] found free-living as well as endosymbiotic methanogens (methanogens living in the cells of Protozoa) in the hindgut of arthropod hosts. Methanogenic Archaea have been detected and their phylogeny analysed in the digestive tracts of methane-producing arthropods other than millipedes by 16S rRNA-based surveys [24], [25], [26], [27], [28]. Moreover, phylogenetic analysis in combination with enrichment cultures has suggested the existence of a seventh order of methanogens (the “Methanoplasmatales”) associated with intestinal tracts of both invertebrates and vertebrates [29]. A detailed assessment of methanogenic diversity is still lacking in millipedes, however, because microbial communities in millipede digestive tracts have mainly been studied only by classical cultivation methods and microscopic observation [19], [30].

Unlike the digestive tract of most methane-producing arthropods, millipedes have a relatively simple digestive tract that lacks an enlarged pouch in the hindgut [19]. The inner surface of the millipede hindgut, however, is strongly developed and may facilitate microbial colonization [30].

According to Hopkin and Read [31], there is no evidence that millipedes have a permanent symbiotic microbiota similar to that of termites, although microorganisms are important for millipede digestion. But the review of Byzov [30] demonstrated that the millipede intestinal tract harbours a stable, indigenous microbial community that includes facultative anaerobes that are able to degrade recalcitrant organic polymers. Only a few studies have used molecular techniques to study the microbial community of millipede faeces [32] or millipede intestines [29], [33].

Specific autofluorescence microscopy showed that the digestive tract of some millipedes contains free methanogenic Archaea or *Nyctotherus*-type ciliates that host methanogens as endosymbionts [19]. van Hoek [34] detected one archaeal sequence obtained from a symbiotic ciliate of an undetermined millipede, while Paul and colleagues [29] detected methanogens related to the “Methanoplasmatales” in the gut of the tropical millipede *Anadenobolus* sp.; no information is available, however, about the differences in intestinal archaeal communities among different millipede lineages.

Methane is known to be produced by almost all tropical millipedes but is generally thought not to be produced by millipedes and cockroaches from temperate climates [19]. Šustr and Šimek [23], however, detected methane production in several species of European millipedes. Their results indicated that some groups of European millipedes (mainly Julida) seem to produce methane, while other groups do not (Polydesmida and Glomerida). Because millipedes apparently include producers as well as non-producers of methane, they represent an interesting model group for the determination of factors influencing gut methanogenesis.

In the current study, we compared methane production and the community structure of methanogens among different phylogenetic lineages of millipedes. In doing so, we attempted to identify factors that explain the phylogenetic differences. To accomplish these objectives, we used gas chromatography in order to measure methane production for a large number of taxonomically diverse millipede species. We verified the presence of methanogens by using PCR to detect the specific genetic marker (*mcrA* gene), and we used molecular profiling (DGGE) to compare the community structure of methanogens in the gut and in faecal pellets of different species of millipedes. Finally, we discuss how methane production relates to millipede phylogeny and millipede characteristics.

## Materials and Methods

### Animals

In the current study, most data used to evaluate methane production relative to millipede taxonomic position were obtained by gas chromatography. These data were supplemented by the data published in the recent literature [19], [23]. The European millipedes used for our measurements were collected in different habitats in the Czech Republic, Slovakia, Romania, and Greece. The localities of origin of the species included in this study are listed in Table S2.

The permission for the research in Tatra Mountains was issued by the Regional Office of Environment Protection, Prešov, Slovakia (permission No. 1/2007/01066-005/KM-R). Millipedes from the Latorica Protected Landscape Area (PLA) were collected with the permission of the Regional Office of Environment Protection, Košice, Slovakia (permission No. 2008/00322), and the research in the Slovak Karst was licensed by the Ministry of the Environment of the Slovak Republic (licence No. 3102/2009-2.1/jam.). The research in the Bohemian Karst PLA was permitted by the Administration of Český kras PLA, Karlštejn, Czech Republic; in the Blanský les PLA by the Administration of Blanský les PLA, Vyšný, Český Krumlov, Czech Republic; in the Moravian Karst PLA by the Administration of Moravský kras PLA, Blansko, Czech Republic; and in the Pálava PLA by the Administration of Pálava PLA, Mikulov, Czech Republic. No specific permission was required to collect millipedes in the following five localities in the Czech Republic: the České Budějovice Basin (49°12′0.793″N, 14°24′25.628″E), the Chelčice (49°06′14.026″N, 14°07′56.443″E), the Českomoravská vrchovina Highlands (49°12′56.873″N, 15°54′55.612″E), the Lanžhot (48°43′6.003″N, 16°58′11.273″E), and the Ždánický les Highlands (49°04′41.946″N, 16°56′10.599″E). None of the species collected in the Czech Republic are endangered or protected in the Czech Republic. The species collected in Romania are also not endangered or protected. No specific permission was required for the sampling in the locality Mehedinţi Mts. (45°04′14.90″S 22°45′48.35″E). The species collected in Thessaloniki (40°36′56.37″N, 23°02′08.96″E) is not endangered or protected in Greece, and no specific permission was required for this location.

Specimens of the millipede *Archispirostreptus gigas*, which is naturally distributed in Tanzania, were obtained from a pet shop (M. Kroček, Horní Suchá, Czech Republic). Two species of millipedes were obtained from a laboratory colony maintained at MPI for Terrestrial Microbiology Marburg (Germany); these species were *Glomeris marginata*, which occurs naturally in Germany, and *Orthomorpha coarctata*, which occurs naturally in Southeast Asia. Specimens of *Epibolus pulchripes*, which occurs naturally in Tanzania, were obtained from a breeding colony maintained by the Mikulov grammar school (Czech Republic).

All other localities mentioned in the Table S2 are cited as sites of origin of species used by Hackstein and Stumm [19].

The millipedes were identified to species based on the available literature; before they were assessed for methane production, the millipedes were kept in the laboratory at 10°C for several days to several weeks in plastic boxes containing top soil and litter from the collection site. Appropriate moisture was maintained by regularly moistening the substrate with water, and cuttlefish bone powder was added to the substrate as a source of calcium. Because the diet of most European species of millipedes consists of decomposed leaf litter, the litter in the boxes served as a food source.

As noted earlier, the tropical species *E. pulchripes* and *A. gigas* were obtained from a pet shop and from individual breeders. Species determination of *A. gigas* was verified based on gonopod morphology after Mwabvu et al. [35]. The tropical species were kept at about 25°C on horticultural substrate plus hardwood leaf litter. *E. pulchripes* accepted partly decomposed litter from hardwood trees (a mixture of hazel, maple, and oak leaves) as the only source of food. *A. gigas* consumed large amounts of organic wastes such as pieces of potatoes, cucumbers, and cabbage heads in addition to leaf litter and garden substrate. Coprophagy of millipede faecal pellets was not prevented for either of the tropical species. In total, 35 species of millipedes were maintained and assessed for methane production. Only individuals taken directly from the substrate and with full digestive tracts were used for gut dissection and subsequent microbial analyses.

### Methane production measurement

Methane production from intact, living millipedes was measured by comparing methane concentrations in closed vessels (250 ml, 5 ml, or 1 ml depending on millipede size) that contained or did not contain millipedes – control vessels. The largest species, which were *A. gigas*, *E. pulchripes*, and the cockroach *Blaptica dubia* (*B. dubia* was used as a known methane producer), were measured in 250-ml glass vessels. Intermediate-sized species such as *Julus scandinavius* were measured in 5-ml glass vessels. The 250- and 5-ml vessels were sealed with a rubber stopper and kept at 20°C for 3–4 h (in the case of 250-ml vessels) or for 5–13 h (in the case of 5-ml vessels). A piece of filter paper (2×4 cm for 250-ml vessels or 1×1 cm for 5 ml vessels) moistened with distilled water was placed in each glass vessel to maintain air humidity during measurement. The control glass vessels without animals were assembled and incubated in the same way. A 0.5-ml sample of the internal gas was collected at the start and at the end of incubation using gas-tight syringes, and the samples were injected into a gas chromatograph (GC) column.

The smallest species were incubated in a 700-µl volume in 1-ml plastic syringes [36]. A circle of moist filter paper (5 mm in diameter) was placed in the syringe to maintain air humidity. Fresh outdoor air was drawn into the syringe immediately before placement of animals. The syringes were incubated at 20°C, and the incubation period ranged from 4 h for the largest species to 24 h for the smallest species in syringes. As controls, the same syringes without animals were assembled, and the mean final concentration of methane in the controls was subtracted from the final concentration in samples. A 500-µl volume of the internal atmosphere in the syringe was injected directly into a GC after the incubation period. The amount of methane was quantified using an HP 5890 Series II gas chromatograph (Hewlett Packard, Palo Alto, CA, USA) equipped with a 2 m Porapak N column at 75°C, and a flame ionization detector using nitrogen as the carrier gas. A standard mixture of 100 ml m^−3^ methane in N_2_ was used for calibration purposes [23].

In most cases, one animal was placed in one glass vessel or plastic vessel (syringe) to detect the individual variability in methane production. For some of the smallest species, for which incubation of individuals resulted in non-detectable methane production, several individuals were incubated in one syringe to verify the negative result (for *T. costata*, for example, three groups of three animals were measured). Animals were weighed at the end of the incubation, and the results were expressed as nl of methane produced per individual per h or as the live-mass specific methane production (nl of methane per g of live mass per h).

The ability to detect methane production depends on the sensitivity of the detection method and on the precision of measurement expressed in the variability of blank controls. If a small amount of methane is generated, the detection limit must be determined to distinguish between negative and positive measurements. We considered a value to be positive if it exceeded the value obtained in five control vessels (glass or plastic vessels without animals) by three standard deviations of controls. In addition, we designated species as non-producers (0% positive samples), accidental producers (at least one but <50% positive samples), facultative producers (from 50% to 99% positive samples), and obligatory producers (100% positive samples).

The distribution of methane-positive species among different taxonomic groups was summarized in contingency tables, and a nonparametric Χ^2^ test (Statistica v6.0 Nonparametric Statistics, 2×2 tables, StatSoft, Inc., USA) was used to test the hypothesis of random distribution of methane production across the millipede phylogenetic tree. We used the previously published taxonomic classification of millipedes [37] along with the phylogenetic tree based on molecular data [38]. The effect of the mean species body mass on the frequency of methane-positive individuals in the species was tested by logit regression in Statistica v6.0 (Advanced Linear/Nonlinear Models: Nonlinear Estimation Analysis).

The faecal pellets of millipedes were anaerobically incubated at 20°C for 7 days to measure their methane production. Fresh pellets were placed in 5-ml glass vessels and moistened with 100 µl of distilled water. The atmosphere in the vessels was changed to argon, and the internal gas was sampled and analysed after 24, 96, and 168 hours by GC as described previously.

### Molecular evaluation of the presence and diversity of methanogenic microorganisms in millipedes

Dissected digestive tract contents or freshly released faecal pellets were frozen and stored at −18°C until DNA extraction. Two methods of DNA extraction were tested. A phenol/chloroform extraction method based on bead-beating homogenization of frozen samples [39] successfully extracted DNA mainly from the faecal pellets or the intestinal tracts of larger animals. For the intestinal tracts of smaller animals (mostly temperate, European millipedes), the NucleoSpin Tissue XS kit (Macherey-Nagel, Germany), based on enzymatic and chemical lyses, enabled the extraction of concentrated DNA from small amounts of starting material (0.025–10 mg). The quantity and quality of extracted DNA was determined with a NanoDrop ND-2000 spectrophotometer (Thermo Scientific, Wilmington, DE, USA).

For detection (expressed as presence or absence) of methanogens in the samples, we used PCR amplification of the *mcrA* gene, which encodes the methyl coenzyme M reductase α-subunit. The *mcrA* gene was amplified with two PCR assays, the results of which were combined for detection of positive signals (*mcrA-1* and *mcrA-2*, Table S1). Both reactions were performed in 25-µl reaction volumes containing 1 µl of diluted DNA as template (usually around 5–10 ng), Qiagen Taq DNA polymerase (1.25 U), dNTPs (0.2 mM each), BSA (0.25 µg, Thermo Scientific, USA), and primers (5 pmols and 8 pmols, respectively) in the presence of MgCl_2_ (1.5 and 2.5 mM, respectively) in the PCR buffer recommended by the manufacturer (1x PCR buffer, Qiagen, CA, USA). A 5-µl quantity of the PCR products was analyzed in a 1% agarose gel (Top Vision agarose, Fermentas) that was stained with ethidium bromide (1 mg L^–1^) for 30 min [40], [ 41].

Methanogenic community structure was evaluated by DGGE using a nested PCR approach that targeted the 16S rRNA gene. In the first round, a stretch of the 16S rRNA gene was amplified using primers Ar109F and Ar915R (both at 25 pmol [42]). In the second round, diluted PCR product (1∶100) served as template for amplification of methanogen-specific 16S rRNA using primers MG0357F-gc and MG0691R (both at 25 pmol [43]). Both reactions were carried out with Taq DNA polymerase (Qiagen, CA, USA) and reaction buffer supplemented with 4 µg of BSA (Thermo Scientific, USA). The cycling conditions for each assay are listed in Table S1. DGGE was performed with an Ingeny PhorU system (Ingeny, Leiden, The Netherlands) as follows: 200 ng of PCR product was loaded on an 8% (w/v) polyacrylamide gel with a denaturing gradient range of 30–60% (100% denaturant is equivalent to 7 M urea and 40% deionized formamide). Electrophoresis was run in 0.5×TAE buffer for 16 h at 60°C and 100 V. The DGGE gels were stained with SYBR Green I nucleic acid stain (Lonza Rockland, ME, USA), and molecular profiles were analyzed with GelCompar II software (Applied Maths, Ghent, Belgium). Bands were excised from gels using a sterilized dissector, eluted in 20 µl of MilliQ water with a freeze-thaw cycle, and re-amplified with primers MG0357F–MG0691R. The resulting PCR products were purified using a MinElute PCR Purification kit (Qiagen, CA, USA) and sequenced using an ABI Prism 3100-Avant Genetic Analyser (Applied Biosystems, CA, USA). The sequences were edited with Bioedit 7.0.4.1 software [44] and assembled in Geneious version Pro 5.5.6 created by Biomatters (New Zealand, available from http://www.geneious.com/). Sequences were tested against the GenBank database (www.ncbi.nml.nih.gov) using the Blastn algorithm. The sequences are available in GenBank under accession numbers KF574048–KF574078 and KF739300–KF739307.

The relationship between the results of molecular detection of methanogens and GC detection of methane production was tested by nonparametric statistical methods (Statistica v6.0 Nonparametric Statistics, 2×2 tables). Differences in the complexity of methanogenic communities (numbers of bands detected by DGGE) among different groups of millipedes were tested with a nonparametric Kruskal-Wallis test or Wilcoxon Matched Pairs test (Statistica v6.0, Nonparametric Statistics).

## Results

### Phylogenetic overview of methane production

Table S2 summarizes the data on the presence or absence of methanogenesis in living and intact millipedes belonging to 46 species and eight orders. Data for 35 species were obtained in the current study, and data for the other 11 species were obtained from the literature [19], [23]. With respect to methane production, the species are designated as non-producers (NP), accidental producers (AP), facultative producers (FP), and obligatory producers (OP). Except for *Glomeris tetrasticha*, which was an accidental producer, all species in the order Glomerida were non-producers according to GC analysis (Table S2). Millipedes in the orders Polyzoniida and Chordeumatida (Table S2) were also non-producers. The order Callipodida was represented by only one individual of *Callipodella fasciata*, which did not produce methane. The order Julida included all categories of methane producers, from non-producers to obligate producers. Of the 18 species tested in the family Julidae, only three were obligatory producers; non-producers, accidental producers, and facultative producers were each represented by five species in the Julidae. Two species were tested in the family Blaniulidae, and neither produced measurable methane. The spirobolid species *E. pulchripes* was designated an obligatory producer, and the same designation probably applies to the other members of the order Spirostreptida. All *A. gigas* tested in this study produced large amounts of methane. Among the millipedes belonging to the order Polydesmida that were tested in this study, only the tropical species *Orthomorpha coarctata* was classified as an obligatory producer (only one individual was tested).

European members of the order Polydesmida belonging to the families Paradoxosomatidae, Polydesmidae, and Trichopolydesmidae did not produce detectable levels of methane except for one of the four tested individuals of *Strongylosoma stigmatosum* (Table S2). The data available for this order are biased, however, because most polydesmid methane producers are tropical and large species (*O. coarctata* and *Pycnotropis acuticollis* [19]) but most of the tested polydesmids were European and small species.

**Figure 1:**
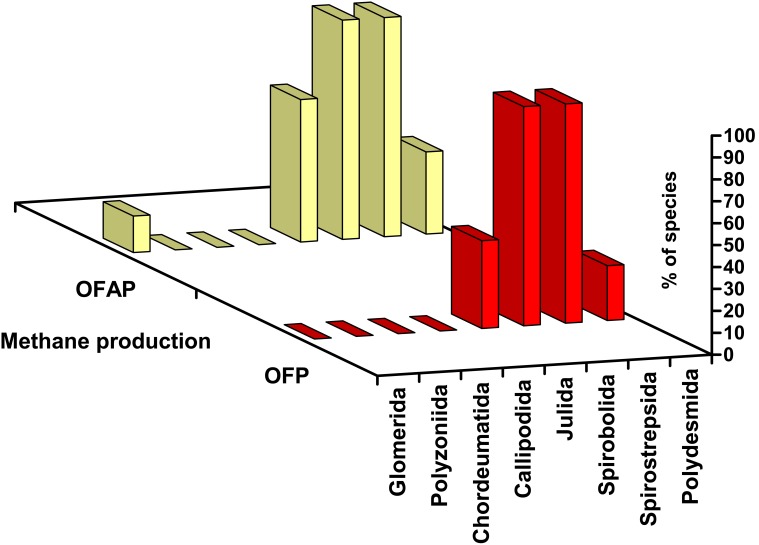
The distribution of methane-positive species among millipede orders. The red row of columns (OFP) is based on all tested species classified as either obligatory or facultative producers, and the yellow row (OFAP) includes obligatory, facultative, and accidental producers.

**Figure 2:**
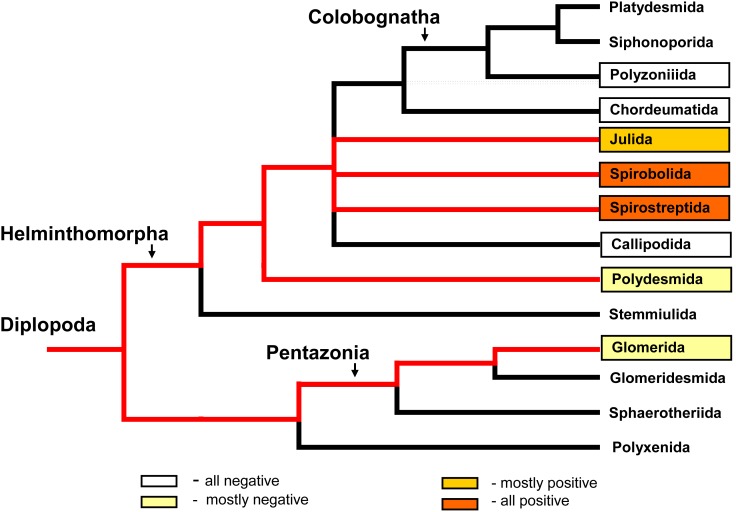
Summary of the recent information concerning methane production by millipedes. Methane production detected by gas chromatography (Table S1) relative to millipede phylogeny. Species belonging to framed groups have been tested to date. Red lines mark the lineages containing some positive species. The phylogenetic tree was used according to Regier et al. [38].

The current data and previously published data [19], [23] were used to describe the distribution of methane-positive species among the main millipede orders (Fig. 1 and 2). The methane-positive species (obligatory and facultative methane producers) are distributed almost exclusively in the juliform groups (the orders Julida, Spirobolida, and Spirostreptida). The order Julida, which was the most investigated group, had a higher percentage of accidental producers and non-producers than the Spirobolida and Spirostreptida. In a comparison of the frequencies of all methane-producing species (AP, FP, and OP together) in the juliform orders Julida, Spirobolida, and Spirostreptida vs. in all other groups, the Χ^2^ test of the 2×2 contingency table indicated that taxonomic position was significantly related to methane production (Χ^2^(df = 1) = 13.61, p = 0.0002).

### Quantitative aspects of methane production

**Figure 3:**
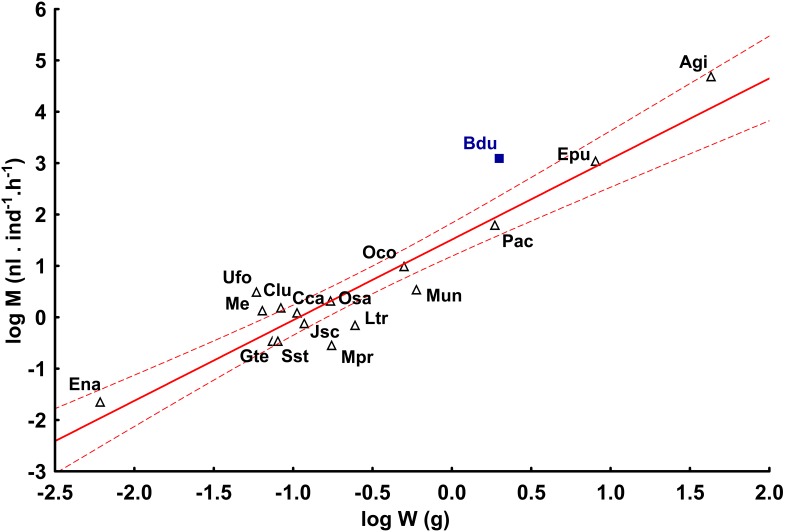
The relationship between individual methane production and the mean body mass. Mean methane production (M) in methane-positive species of millipedes (accidental, obligatory, and facultative producers) plotted against mean body mass of species (W). Species abbreviations are indicated in Table S1. Methane production by the cockroach *Blaptica dubia* (Bdu) is included for comparison. Red lines = regression of M on W with 95% confidence intervals.

One adult individual of the millipede *A. gigas* produced more methane than the smaller tropical *E. pulchripes* in the order Spirobolida and several orders of magnitude more than small European species in the order Julida. The mean individual methane production from *A. gigas* is comparable to the methane production from the large cockroach, *Blaptica dubia*. Mean individual methane production (M) by species was positively related to average live body mass (W) by species as indicated by a double logarithmic plot (Fig. 3). The linear regression of log M on log W corresponded to the equation M = 32.494·W^1.569^ (r^2^ = 0.899, p = 0.0001). Regression of body mass-specific methane production (M/W) on millipede mass (W) was also positive and significant (r^2^ = 0.522, p = 0.002). It follows that, in addition to taxonomic position, body size may explain differences in methane production among millipede species.

We also analyzed changes in the percentages of methane-positive and methane-negative individuals with decreasing mean body mass in an attempt to detect a general lower limit in body mass for methane production by millipedes. All five species whose body mass was >0.4 g were obligatory producers (OP). The intermediate group in terms of body mass (from 0.06 to 0.30 g) included eight species representing all four categories of methane producers (two species of OP, two species of FP, one species of AP, and three species of NP). All species whose body mass was <0.06 g were non-producers except for *Unciger foetidus* (the mean body mass of the tested individuals was 0.059 g) and the very small julid species *Enantiulus nanus* (its mean body mass was 0.006 g); in the case of *E. nanus*, only one of the 10 tested individuals was positive. In the order Glomerida, the largest species (0.28 g) was classified as NP, and the data set is very small. These data are also scarce for the order Polydesmida, but the existence of some body mass limit for methane production may be expected in this group because all species that were <0.08 g produced no methane and one species that was >0.4 g produced methane. For the juliform orders (Julida, Spirostreptida, and Spirobolida), a body mass limit of 0.12 g separated the methane-positive and methane-negative species.

### Methane production from faecal pellets of millipedes

**Figure 4:**
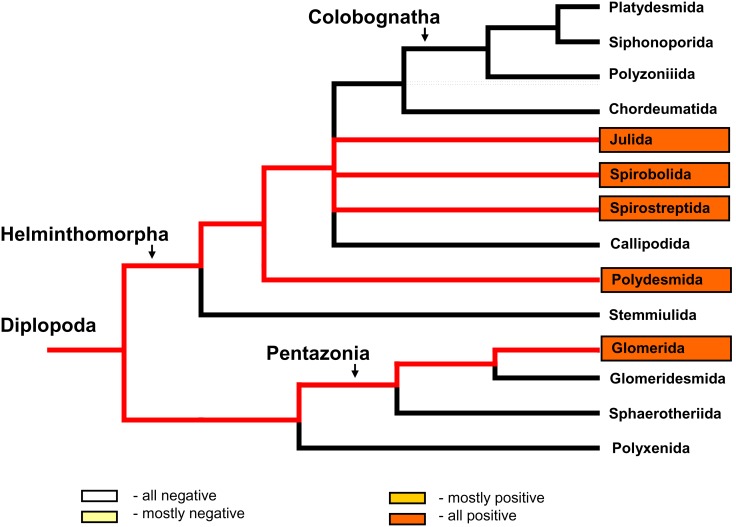
Methane production from millipede faecal pellets relative to millipede phylogeny. Methane production from anaerobically incubated faecal pellets was detected by gas chromatography (Table S1). Species belonging to framed groups were tested in this study. Red lines mark the lineages containing some positive species. The phylogenetic tree was used according to Regier et al. [38].

**Figure 5:**
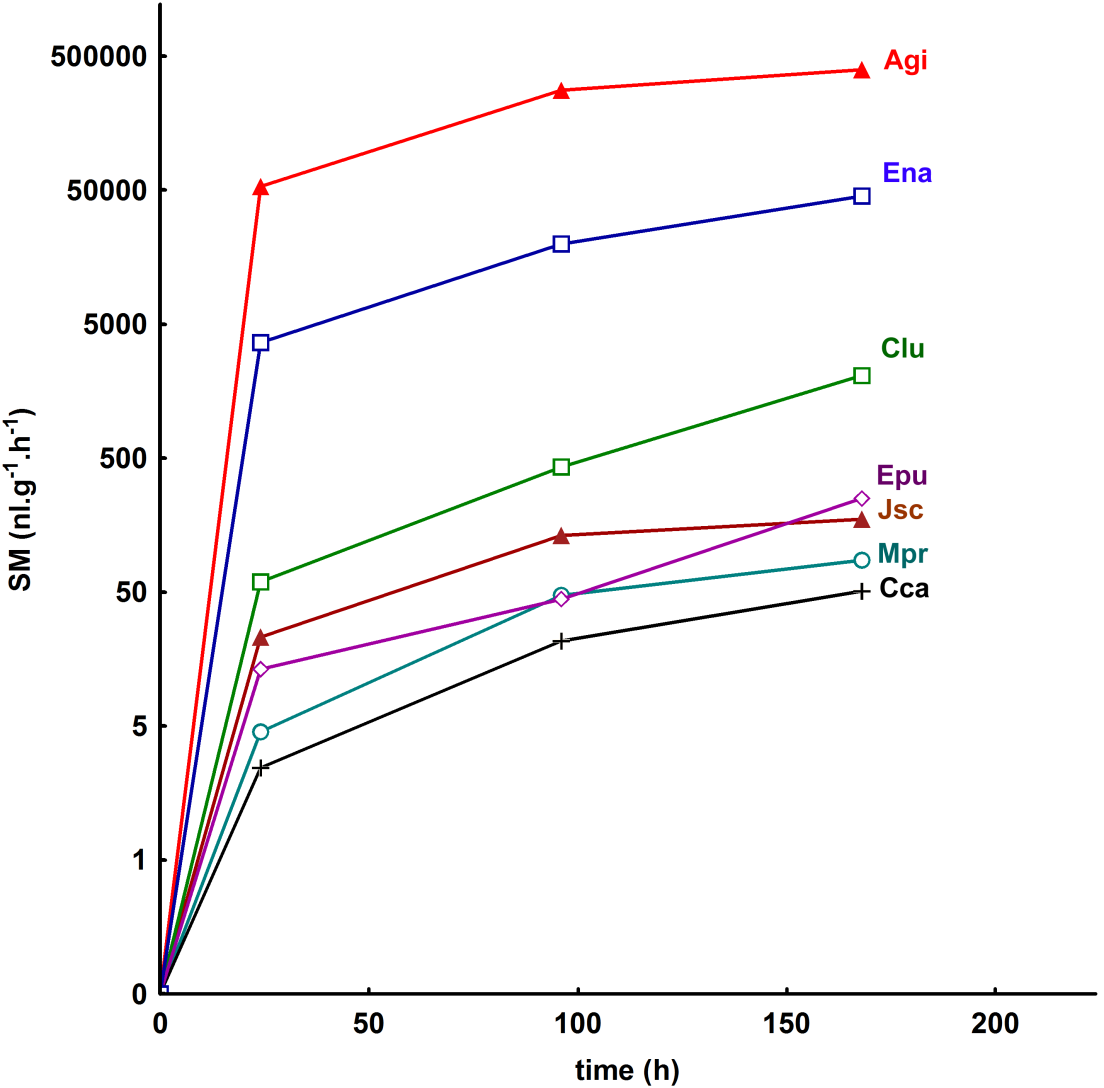
Change in the rate of methane production per mass of millipede faecal pellets over time. The pellets were anaerobically incubated at 20°C. Abbreviations of species are indicated in Table S1.

Excrements of all tested millipede species produced methane under anaerobic conditions (Fig. 4). The time course of the production was similar in all species in that the rate of production quickly increased during the first day of anaerobic incubation and then slowly increased over the next 6 days (Fig. 5).

### Methanogenic community structure

DNA was successfully extracted from the faecal pellets of 16 millipede species and from the gut contents of 13 millipede species (Table S2). The extraction was less effective from gut contents than from faecal pellets. Specific amplification of the *mcrA* gene using two different PCR assays confirmed the presence of methanogens in the gut of three species of millipedes belonging to the orders Julida, Spirostreptida, and Spirobolida. Results were similar for both assays except for *Cylindroiulus luridus* excrements and *Julus scandinavius* gut contents (Table S2), which can be influenced by primer sequence bias. The *mcrA* gene was not found in the gut contents of two species in the family Glomeridae or in the gut contents of the one species in the order Polydesmida (Table S2). A Chi-square test calculated from 2×2 contigency tables indicated that the production of methane as detected by GC was significantly related to the detection of *mcrA* in the gut contents of the same animal (Χ^2^(df = 1) = 4.05, p = 0.044, n = 24). Detection of the *mcrA* gene in gut contents supported the classification of species based on GC measurement of methane production. All three of the positive gut samples originated from species that were designated FP or OP.

**Figure 6:**
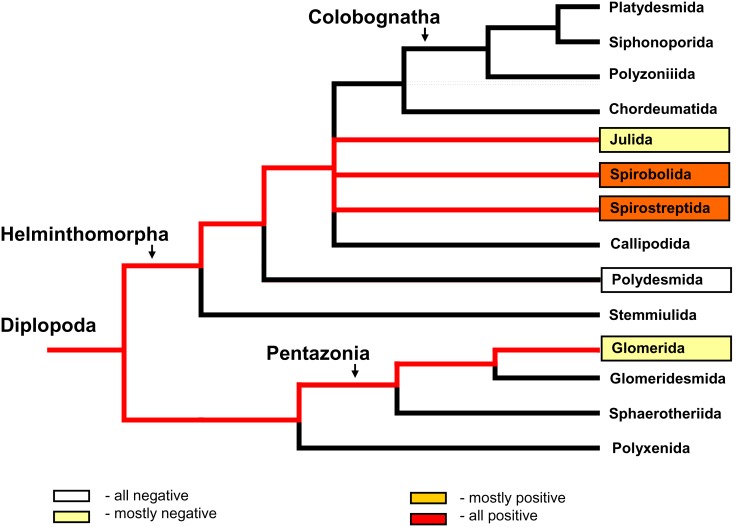
Detection of the archaeal *mcrA* gene in millipedes relative to millipede phylogeny. *mcrA*, the marker gene of methanogenic Archaea, was detected by PCR amplification (Table S1) in gut and faecal pellets of millipedes. Species belonging to framed groups were tested in this study. Red lines mark the lineages containing some positive species. Only faecal pellets were positive in Glomerida. The phylogenetic tree was used according to Regier et al. [38].

For most species, the detection of *mcrA* in the gut contents corresponded to *mcrA* detection in faecal pellets. Discrepancies occurred in only three cases (two in which *mcrA* was detected only in faecal pellets and one in which *mcrA* was detected only in the gut contents). All of the tested millipede faecal pellets began to produce methane early during the incubation under anaerobic, moist conditions but the *mcrA* gene was detected in the faecal pellets of only five of the 16 species that were tested. The detection of *mcrA* in different millipede lineages is illustrated in Figure 6.

**Figure 7:**
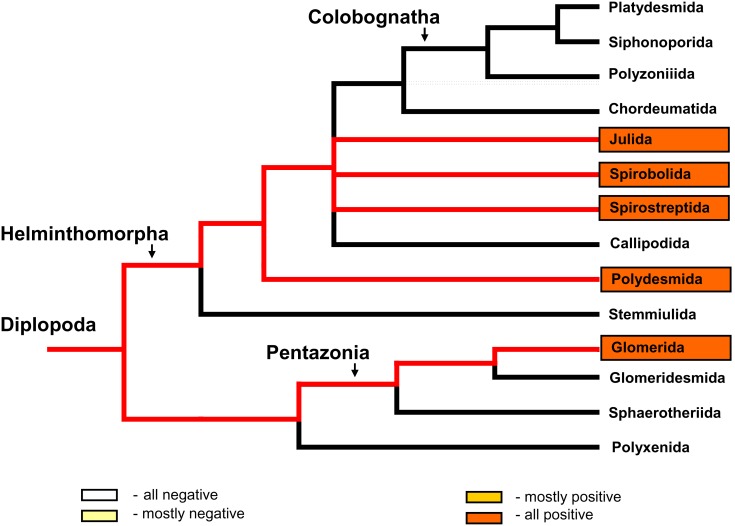
Detection of the archaeal 16S rRNA gene in millipedes relative to millipede phylogeny. The 16S rRNA gene, an indicator of methanogenic Archaea, was detected by PCR amplification (Table S1). Species belonging to framed groups were tested in this study. Red lines mark the lineages containing some positive species. In Polydesmida, only faecal pellets were tested. The phylogenetic tree was used according to Regier et al. [38].

**Figure 8:**
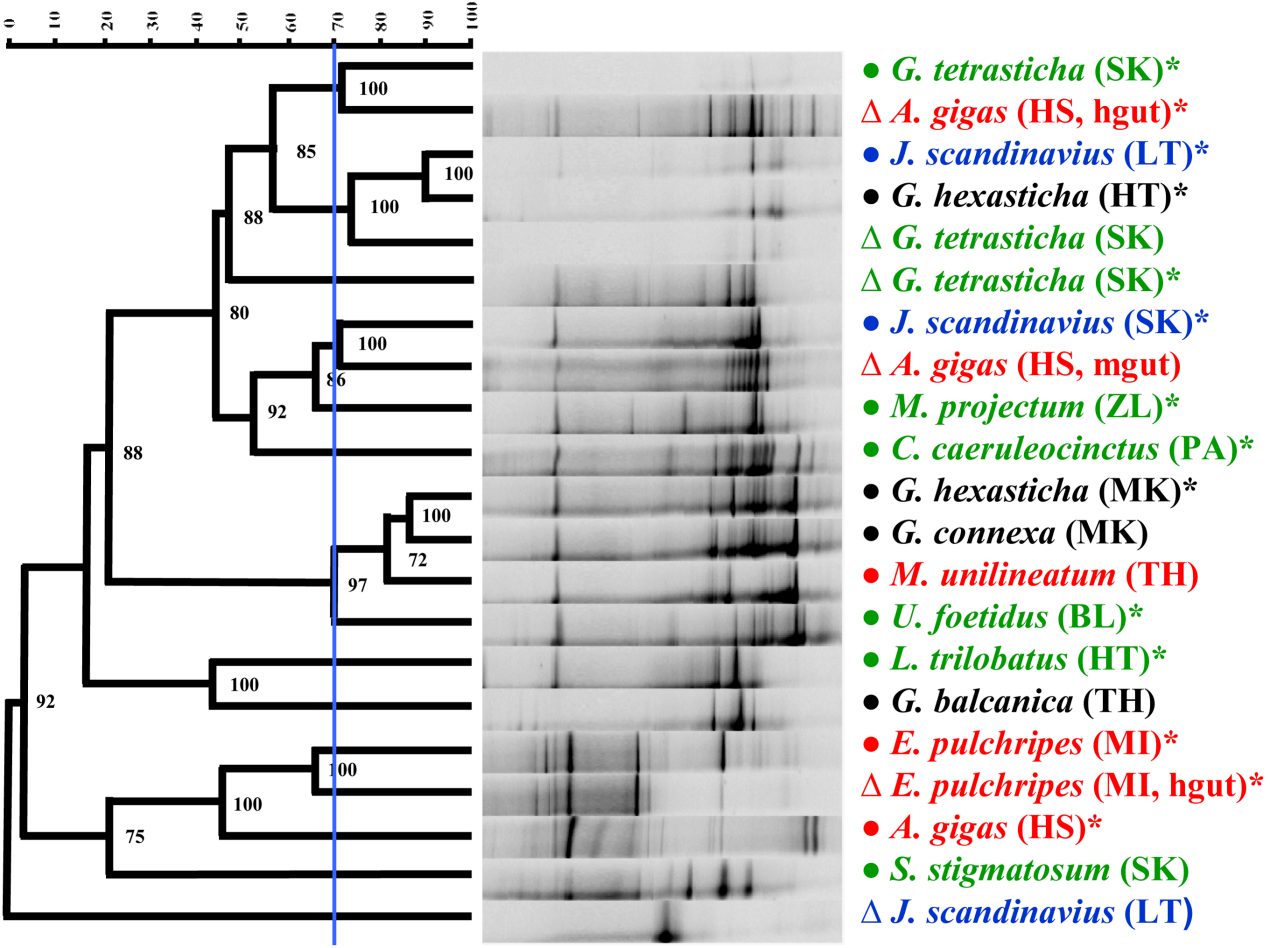
Cluster analysis of methanogenic communities in millipedes. DGGE patterns were obtained after PCR amplification of the 16S rRNA gene of the methanogenic communities in the gut contents (Δ) or faecal pellets (•) of individual millipedes from the indicated species. Pearson correlation and UPGMA analysis were used. Abbreviations in brackets indicate the origin of animal samples (see Table S1). mgut = midgut and hgut = hindgut samples. Species belonging to different pre-defined groups of methane producers are marked with different colours: NP – black, AP – green, FP – blue, and OP – red. Asterisks indicate that the samples were from individuals or faecal pellets in which methane production was detected using gas chromatography. Blue line = similarity cutoff 70%.

DGGE profiles of the methanogenic microbial communities were obtained from 18 samples of the digestive tracts representing 10 species. This method revealed the presence of methanogens in the digestive tract of members of the orders Glomerida, Julida, Spirobolida, and Spirostreptida and in all categories of methane production except NP. Molecular profiles of the methanogenic microbial communities were also obtained from 30 samples of faecal pellets representing 15 species. In addition, methanogens were detected in the faecal pellets of Polydesmida members. The DGGE analysis revealed the presence of the methanogen-specific 16S rRNA gene in all of the tested millipede orders (Fig. 7, Table S2). DGGE analysis also revealed large variability in the methanogenic community structure among different gut and faecal pellet samples in millipedes from the orders Glomerida and Julida. Clustering of molecular profiles (Fig. 8) showed no relation of methanogenic community structure to the pre-defined groups NP, AP, FP, and OP. The results revealed a high diversity of the methanogenic community among millipede individuals, because only a few of the molecular profiles were similar (at similarity cutoff = 70%). The molecular profiles of methanogens from the faecal pellets of *G. hexasticha* (MK), *G. connexa* (MK), *M. unilineatum* (TH), and *U. foetidus* (BL) were similar and significantly different from that of *G. hexasticha* (HT) and *G. tetrasticha* (SK). The methanogenic communities of guts and excrements of the same species were similar in some cases. In other species (*J. scandinavius* and *A. gigas*) the gut and faecal pellet communities formed different clusters. The highest dissimilarity was found between samples of *J. scandinavius*. Methanogenic communities of the whole gut content were almost identical among individuals of *G. tetrasticha* originating from the same locality, but methanogens in the different gut compartments of *A. gigas* had only 40% similarity (Fig. 8).

As indicated by the number of bands in the DGGE profile, some species harboured rich methanogenic communities while others did not. Overall, more DGGE bands were obtained in faecal pellets than in gut contents. This trend was confirmed by nonparametric statistical tests comparing all DGGE profiles obtained from guts and faecal pellets (Kruskall-Wallis test, H (1, n = 48) = 3.99, P = 0.046) and comparing species averages for pellets and guts (Wilcoxon pair test, n = 9, P = 0.038). The number of bands in DGGE profiles was greater for the tropical millipedes (Spirobolida and Spirostreptida), which included the two largest species in this study, than for the European millipedes (Kruskall-Wallis test, H (1, n = 48) = 6.95, P = 0.008) (Fig. 8).

It was not possible to re-amplify and characterize all of the excised bands from each profile, perhaps because of PCR inhibition or a low concentration of the DNA fragment in the eluate. A total of 32 bands, representing specific sequences in the DGGE profiles, were successfully sequenced and identified (Table S3), and these were assumed to represent the specific or dominant components of the communities.

In general, diverse methanogens were identified in the gut and faecal pellets. These included *Methanosarcina thermophila*, *M. siciliae*, *Methanoregula boonei*, *Methanobrevibacter woesei*, *M. thaueri*, and *M. arboriphilus*, as well as other species of these genera that remained unidentified. Methanogenic DGGE profiles obtained from guts and faecal pellets of millipedes belonging to the order Julida were dominated by sequences related to Methanomicrobiales and Methanosarcinales. *Methanoregula* and other unclassified Methanomicrobiales or *Methanosarcina* were often identified in these samples.

In samples representing the millipede family Glomeridae, we found Methanomicrobiales, Methanosarcinales, as well as Methanobacteriales (*Methanobacterium* sp. or *Methanobrevibacter* sp.). In faecal pellets and gut content samples from *S. stigmatosum* (Polydesmida), *E. pulchripes* (Spirobolida), and *A. gigas* (Spirostreptida), only *Methanobrevibacter* spp. and related Methanobacteriales were among the dominant bands that were succesfully identified.

## Discussion

The results presented here show that methane production and the *mcrA* gene are unequally distributed among phylogenetic lineages of millipedes (see Fig. 2 and Fig. 6). Previous research also revealed considerable variation in the presence of methane production among different groups of arthropods, and these differences were thought to reflect differences in the ability to host methanogenic microorganisms [19].

Although detection of methane production and the *mcrA* gene indicate a rather restricted distribution of methanogens in millipedes, we found the 16S rRNA genetic marker of methanogens in all tested taxa (members of Julida, Glomerida, Polydesmida, Spirobolida, and Spirostreptida) and potential anaerobic methane production in all of the tested faecal pellets (see Fig. 7 and Fig. 4). Thus, the methanogens were present in both of the main groups of millipedes: the Helminthophora and Pentazonia. In the current study, the presence of methanogens in the Pentazonia was indicated by methanogenesis in all of their tested faecal pellets, by the detection of methane production from some of living millipedes, and by the detection of methanogen genes. In a previous study, the presence of methanogens in the Pentazonia was supported indirectly by the detection of hydrogen production in the gut of some species of Glomerida [19]. The current results indicate that methanogens may be more broadly distributed in millipedes than previously assumed.

We obtained molecular evidence of methanogens by assessing the ribosomal marker (16S rRNA) and *mcrA* gene. The results obtained with 16S rRNA frequently differed from those obtained with *mcrA*. Methane production was better correlated with detection of *mcrA* than with detection of 16S rRNA. The *mcrA*-based approach may be less sensitive because its primers are more specific than in the 16S rRNA-based approach. The latter feature has been discussed previously [45] and can be explained by a higher gene copy number of the 16S rRNA gene than of the *mcrA* gene in the genomes.

The disagreement between detection of methane production and the molecular evidence of methanogens (based on the 16S rRNA gene) can be attributed to the fact that the presence of genetic markers is not an accurate indicator of activity and may represent only the potential for activity. It also suggests that a very small or inactive methanogenic community may be present in the gut of most millipedes. Such communities might be activated when conditions become favourable. This idea is supported by the ability of methanogens to survive unfavourable conditions (drought and aeration) for long periods [46] and to be activated once anoxic conditions return [47], [48]. Our results indicate that the striking differences in methane production among different millipede taxa reflect differences in the activity of intestinal methanogens rather than an absolute inability of some taxa to host methanogens.

Hackstein and Stumm [19] considered possible sources of variation in the activity of intestinal methanogens, resulting in differences in methane production among arthropod individuals. The authors discussed body size, environmental temperature or climatic zone of the species distribution, number of intestinal protozoa, and unknown genetically fixed factors.

Among the millipedes investigated in our study, the number of methane-producing animals increased with increasing body size of the species. Moreover, the amount of methane produced was positively correlated with millipede body size in methane-producing species. This effect of body size may be explained by the better aeration in the gut of small vs. large millipedes (the gut of smaller animals has a higher surface-to-volume ratio); the increased aeration in the gut of small animals may prevent the proliferation of anaerobic methanogenic Archaea [49]. Although the oxygen concentration in the gut of millipedes has rarely been measured, the mean redox potential in the gut of the pill millipede, *G. marginata* (body mass about 0.2 g), was +232 mV in the midgut and +204 mV in the hindgut, and these values correspond to oxidative conditions [50]. This is consistent with our failure to detect methane production in this species.

Thus, differences in body size may explain much of the between-species variability in methane production of millipedes. The absence of methane production in relatively larger members of the Polyzoniida and Callipodida may be caused by their unique feeding habits. Polyzoniida consume moist, soil detritus [51], and Callipodida contains species that have predatory feeding habits and species that consume the dead tissues of animals [52], [53].

Hackstein and Stumm [19] speculated that methane production would be more common among tropical than temperate species of arthropods. The effect of climatic origin of species may be masked by the effect of body size because, like other terrestrial invertebrates, millipedes tend to be larger in the tropics than in other regions [54]. Although methane production from individual millipedes decreased with decreasing temperature [23], the inference that high environmental temperatures are required for methanogenesis was disproved by the evidence of methane production from European species of the family Julidae [23] as well as by methane production in permafrost soils [55].

Another possible source of the differences in methane production between tropical and temperate taxa may lie in the symbiotic interaction of large tropical millipedes with ciliate protists related to the genus *Nyctotherus* [19]. The ciliates host considerable numbers of methanogens near hydrogen-producing hydrogenosomes [56], [57], where the methanogens consume hydrogen. Therefore, the abundance of ciliates enhances the methanogenic activity and enhances the rate of methane production from cockroaches [17]. It follows that protists may be responsible for quantitative differences in the methane production between tropical spirostreptid millipedes and European Julidae. This is supported by our finding that mainly hydrogenotrophic [58] Methanobacteriales (*Methanobrevibacter arboriphilus* and other *Methanobrevibacter*-related sequences) dominated the gut of large members of the Spirostreptida (*A. gigas*); hydrogenotrophic Methanobacteriales were previously found in the cytoplasm of ciliates of the genus *Nyctotherus* [17], [34]. Moreover, we observed ciliates in the hindgut and fresh faecal pellets of *A. gigas* (unpublished data).

Most methanogenic Archaea lineages previously reported from the insect gut (Methanosarcinales, Methanobacteriales, and Methanomicrobiales; see [59] for a summary) were identified in the present study of millipedes. The list of methanogenic lineages identified here may be completed only by the previously published evidence of the Thermoplasmatales-related methanogens in the hindgut of the millipede *Adenobolus* sp. (Spirobolida) [29] and the DNA sequence of an endosymbiotic methanogen (probably related to the Methanobacteriales) isolated from the ciliate *Nyctotherus velox* from the gut of a “julid” (probably spirostreptid) millipede [34]. The comparison of methanogenic lineages identified in the digestive tracts of larger tropical orders (Spirostreptida and Spirobolida) vs. smaller temperate orders (Julida, Glomerida) of millipedes generated a pattern similar to that obtained for termites [59]. Methanogens colonizing the hindgut of lower termites (which have gut flagellates) and the gut of Spirostreptida millipedes (which have symbiotic ciliates) belong almost exclusively to the genus *Methanobrevibacter*. Methanogens colonizing the hindgut of higher termites are more diverse than those colonizing lower termites and include Methanomicrobiales, Methanosarcinales, and Methanobacteriales, which were the same orders detected in Julida millipedes.

The main lineages of methanogenic Archaea found in different millipede orders differ in their substrate preference and free vs. endosymbiotic mode of life. Therefore, the composition of methanogen communities may reflect differences in the habitats and substrates provided by different lineages of millipedes. *Methanobrevibacter* species isolated from different habitats almost exclusively used H_2_ and CO_2_ as substrates [59]. Some *Methanobrevibacter* phylotypes may be associated with flagellates [60] or ciliates [57], [34]. The metabolism of the strain MPM2, which was identified in spirobolids, resembles H_2_-requiring methylotrophic methanogens [29]. Regarding Methanosarcinales representatives, rather than finding *Methanomicrococcus blatticola*, which is specialized in hydrogen-dependent reduction of methanol or methylamines to methane [22] and which is found in cockroaches and higher termites [59], we found several *Methanosarcina* lineages in julid millipedes. Methanosarcinales are able to use all three of the known metabolic pathways for methanogenesis (hydrogenotrophic, acetoclastic, and methylotrophic) [61] and thus possess a versatile metabolism that is able to respond to environmental change. Additional detailed studies of selected millipede species are needed to determine whether the metabolic plasticity of different lineages of methanogenic Archaea influences which millipede taxa they inhabit.

In conclusion, the qualitative and quantitative variability in methane production among millipedes seems to reflect the suppression or activation of methanogenic Archaea in the digestive tracts of some phylogenetic millipede lineages rather than a fundamental inability of these millipede lineages to host methanogens. Our results also indicate that methane-producing millipede taxa differ in the functional groups of methanogens that they host.

## Supporting Information

Table S1 **PCR protocols.** Primers and PCR conditions used for amplification of the *mcrA* gene and the 16S rRNA gene for DGGE analysis.
(PDF)

Table S2 **Methane production in millipedes and methanogenic microorganisms detected in millipede gut contents and faecal pellets.** Methane production was measured by gas chromatography (GC) in the current study or in previous studies, as indicated. The *mcrA* gene was used as a marker for methanogens in the gut contents and faecal pellets. The presence of methanogens in the gut contents and faecal pellets was also determined by DGGE of the methanogen 16S rRNA gene. Mean values for richness of DGGE profiles are given.
(PDF)

Table S3 **The sequenced bands of methanogenic archaeal genes amplified from millipedes.** Phylogenetic relationships of the excised and sequenced bands of methanogenic archaeal 16S rRNA genes amplified from millipede faecal pellets or gut contents. Sequences that were not submitted to GenBank were those shorter than 200 bp.
(PDF)

## References

[1] Ehhalt D, Prather M, Dentener F, Derwent R, Dlugokencky E, et al.. (2001) Atmospheric Chemistry and Greenhouse Gases. In: Houghton JT, Ding Y, Griggs DJ, Noguer M, van der Linden PJ, Dai X, Maskell K, Johnson CA, editors. Climate Change 2001: The Scientific Basis. Contribution of Working Group I to the Third Assessment Report of the IPCC. Cambridge, United Kingdom and New York: Cambridge University Press. 239–287.

[2] Ramaswamy V, Boucher O, Haigh J, Hauglustaine D, Haywood J, et al.. (2001) Radiative Forcing of Climate Change. In: Houghton JT, Ding Y, Griggs DJ, Noguer M, van der Linden PJ, Dai X, Maskell K, Johnson CA, editors. Climate Change 2001: The Scientific Basis. Contribution of Working Group I to the Third Assessment Report of the IPCC. Cambridge, United Kingdom and New York: Cambridge University Press. 349–416.

[3] EPA (2010) Methane and nitrous oxide emissions from natural sources Washington. EPA 430-R-10-001. U.S. Environmental Protection Agency. 194p.

[4] Dlugokencky EJ, Nisbet EG, Fischer R, Lowry D (2011) Global atmospheric methane: budget, changes and dangers. Philos. T. Roy. Soc. A 369: 2058–2072. 10.1098/rsta.2010.0341 21502176

[5] Ehhalt DH (1974) The atmospheric cycle of methane. Tellus 26: 58–70.

[6] Garcia JL, Patel BKC, Ollivier B (2000) Taxonomic, phylogenetic, and ecological diversity of methanogenic Archaea. Anaerobe 6: 205–226. 16887666 10.1006/anae.2000.0345

[7] Eckburg PB, Bik EM, Bernstein ChN, Purdom E, Dethlefsen L, et al (2005) Diversity of the human intestinal microbial flora. Science 308: 1635–1638. 15831718 10.1126/science.1110591 PMC1395357

[8] Liu Y, Whitman WB (2008) Metabolic, phylogenetic, and ecological diversity of the methanogenic archaea. Ann. NY. Acad. Sci. 1125: 171–189. 10.1196/annals.1419.019 18378594

[9] Kušar D, Avguštin G (2010) Molecular profiling and identification of methanogenic archaeal species from rabbit caecum. FEMS Microbiol. Ecol. 74: 1–8. 10.1111/j.1574-6941.2010.00980.x 20950344

[10] Crutzen PJ, Aselmann I, Seiler W (1986) Methane production by domestic animals, wild ruminants, other herbivorous fauna, and humans. Tellus 38B: 271–284.

[11] Moss AR, Jouany JP, Newbold J (2000) Methane production by ruminants: its contribution to global warming. Ann. Zootech. 49: 231–253.

[12] Makkar HPS, Vercoe PE (2007) Measuring methane production from ruminants. Dordrecht: Springer. 138 p.

[13] Wilkinson JM (2012) Methane production by ruminants. Livestock 17: 33–35.

[14] Breznak JA (1982) Intestinal microbiota of termites and other xylophagous insects. Annu. Rev. Microbiol. 36: 323–343. 10.1146/annurev.mi.36.100182.001543 6756291

[15] Rasmussen RA, Khalil MAK (1983) Global production of CH_4_ from termites. Nature 301: 700–702.

[16] Cruden DL, Markovetz AJ (1987) Microbial ecology of the cockroach gut. Annu. Rev. Microbiol. 41: 617–643. 10.1146/annurev.mi.41.100187.003153 3318681

[17] Gijzen HJ, Broers CAM, Barugahare M, Stumm CK (1991) Methanogenic bacteria as endosymbionts of the ciliate *Nyctotherus ovalis* in the cockroach hindgut. Appl. Environ. Microbiol. 57: 1630–1634. 10.1128/aem.57.6.1630-1634.1991 PMC183443 1908205

[18] Jamali H, Livesley SJ, Dawes TZ, Cook GD, Hutley LB, Arndt SK (2011) Diurnal and seasonal variations in CH_4_ flux from termite mounds in tropical savannas of the Northern Territory, Australia. Agr. Forest Meteorol. 151: 1471–1479.

[19] Hackstein JHP, Stumm CK (1994) Methane production in terrestrial arthropods. Proc. Natl. Acad. Sci. USA 91: 5441–5445. 10.1073/pnas.91.12.5441 PMC44011 8202505

[20] Rosenberg J, Hackstein JHP (1995) Methanbildende Blatthornkäfer (Scarabaeidae, Coleoptera), In: Löser, S. editor. Verhandlugen Westdeutscher Entomologentag, Düsseldorf: Löbbecke-Museum. 67–72.

[21] Bijnen FGC, Harren FJM, Hackstein JHP, Reuss J (1996) Intracavity CO laser photoacoustic trace gas detection: cyclic CH_4_, H_2_O and CO_2_ emission by cockroaches and scarab beetles. Appl. Optics 53: 5357–5368. 10.1364/AO.35.005357 21127531

[22] Sprenger WW, van Belzen MC, Rosenberg J, Hackstein JHP, Keltjens JT (2000) *Methanomicrococcus blatticola* gen. nov., sp. nov., a methanol- and methylamine-reducing methanogen from the hindgut of the cockroach *Periplaneta americana*. Int. J. Syst. Evol. Microbiol. 50: 1989–1999. 10.1099/00207713-50-6-1989 11155972

[23] Šustr V, Šimek M (2009) Methane release from millipedes and other soil invertebrates in Central Europe. Soil Biol. Biochem. 41: 1684–1688.

[24] Ohkuma M, Noda S, Horikoshi K, Kudo T (1995) Phylogeny of symbiotic methanogen in the gut of termite *Reticulitermes speratus*. FEMS Microbiol. Lett. 134: 45–50. 10.1111/j.1574-6968.1995.tb07912.x 8593954

[25] Ohkuma M, Noda S, Kudo T (1999) Phylogenetic relationships of symbiotic methanogens in diverse termites. FEMS Microbiol. Lett. 171: 147–153. 10.1111/j.1574-6968.1999.tb13425.x 10077839

[26] Hara K, Shinzato N, Seo M, Oshima T, Yamagishi A (2002) Phylogenetic analysis of symbiotic Archaea living in the gut of xylophagous cockroaches. Microbes Environ. 17: 185–190.

[27] Egert M, Wagner B, Lemke T, Brune A, Friedrich MW (2003) Microbial community structure in midgut and hindgut of the humus-feeding larva of *Pachnoda ephippiata* (Coleoptera: Scarabaeidae). Appl. Environ. Microbiol. 69: 6659–6668. 10.1128/AEM.69.11.6659-6668.2003 PMC262301 14602626

[28] Dighe AS, Jangid K, Gonzalez JM, Pidiyar VJ, Patole MS, et al (2004) Comparison of 16S rRNA gene sequences of genus *Methanobrevibacter*. BMC Microbiol. 4: 20. 10.1186/1471-2180-4-20 PMC415545 15128464

[29] Paul K, Nonoh JM, Mikulski L, Brune A (2012) “Methanoplasmatales”. Thermoplasmatales-related archaea in termite guts and other environments, are the seventh order of methanogens. Appl. Environ. Microbiol. 78: 8245–8253. 10.1128/AEM.02193-12 PMC3497382 23001661

[30] Byzov BA (2006) Intestinal microbiota of millipedes, In: König H, Varma A, editors, Intestinal microorganisms of termites and other invertebrates. Soil Biology 6, Berlin, Heidelberg: Springer Verlag. 89–114.

[31] Hopkin SP, Read HJ (1992) Biology of millipedes. New York: Oxford University Press. 233p.

[32] Oravecz O, Nyiro G, Marialigeti K (2002) A molecular approach in the analysis of the faecal bacterial community in an African millipede belonging to the family Spirostreptidae (Diplopoda). Eur. J. Soil Biol. 38: 67–70.

[33] Knapp BA, Seeber J, Podmirseg SM, Rief A, Meyer E, et al (2009) Molecular fingerprinting analysis of the gut microbiota of *Cylindroiulus fulviceps* (Diplopoda). Pedobiologia 52: 325–336.

[34] van Hoek AHAM, van Alen TA, Sprakel VSI, Leunissen JAM, Brigge T, et al (2000) Multiple acquisition of methanogenic archaeal symbionts by anaerobic ciliates. Mol. Biol. Evol. 17: 251–258. 10.1093/oxfordjournals.molbev.a026304 10677847

[35] Mwabvu T, Hamer M, Slotow R, Barraclough D (2010) A revision of taxonomy and distribution of *Archispirostreptus* Silvestri 1895 (Diplopoda, Spirostreptida, Spirostreptidae), and description of a new *Spirostreptus* genus with three new species. Zootaxa 2567: 1–49.

[36] Šimek M, Šustr V (1995) Gas chromatographic microrespirometry: Further improvement and application in animal ecophysiology. Soil. Biol. Biochem. 27: 1227–1229.

[37] Tree of Life Web Project. 2002. Diplopoda. Millipedes. Version 01 January 2002 (temporary). Available: http://tolweb.org/Diplopoda/2532/2002.01.01. Accessed 2014 March 11.

[38] Regier CJ, Wilson MH, Shultz JW (2005) Phylogenetic analysis of myriapoda using tree nuclear protein-coding genes. Mol. Phylogenet. Evol. 34: 147–158. 10.1016/j.ympev.2004.09.005 15579388

[39] Griffiths RI, Whiteley AS, O'Donnell AG, Bailey MJ (2000) Rapid method for coextraction of DNA and RNA from natural environments for analysis of ribosomal DNA- and rRNA-based microbial community composition. Appl. Environ. Microbiol. 66: 5488–5491. 10.1128/aem.66.12.5488-5491.2000 PMC92488 11097934

[40] Luton PE, Wayne JM, Sharp RJ, Riley PW (2002) The mcrA gene as an alternative to 16S rRNA in the phylogenetic analysis of methanogen populations in landfill. Microbiology 148: 3521–3530. 12427943 10.1099/00221287-148-11-3521

[41] Kim SY, Lee SH, Freeman C, Fenner N, Kang H (2008) Comparative analysis of soil microbial communities and their responses to the short-term drought in bog, fen, and riparian wetlands, Soil Biol. Biochem. 40: 2874–2880.

[42] Lueders T, Manefield M, Friedrich MW (2004) Enhanced sensitivity of DNA- and rRNA-based stable isotope probing by fractionation and quantitative analysis of isopycnic centrifugation gradients. Environ. Microbiol. 6: 73–78. 10.1046/j.1462-2920.2003.00536.x 14686943

[43] Watanabe T, Asakawa S, Nakanuta A, Nagaoka K, Kimura M (2004) DGGE method for analyzing 16S rDNA of methanogenic archaeal community in paddy field soil. FEMS Microbiol. Lett. 232: 153–163. 10.1016/S0378-1097(04)00045-X 15033234

[44] Hall TA (1999) Bioedit: a user-friendly biological sequence alignment editor and analysis program for Windows 95/98/NT. Nucleic Acids Symposium Series 41: 95–98.

[45] Koubová A, Goberna M, Šimek M, Chroňáková A, Pižl V, et al (2012) Effects of the earthworm *Eisenia andrei* on methanogens in a cattle-impacted soil: A microcosm study. Eur. J. Soil Biol. 48: 32–40.

[46] Mayer HP, Conrad R (1990) Factors influencing the population of methanogenic bacteria and the initiation of methane production upon flooding of paddy soil. FEMS Microbiol. Ecol. 73: 103–112.

[47] Peters V, Conrad R (1995) Methanogenic and other strictly anaerobic-bacteria in desert soil and other oxic soils. Appl. Environ. Microbiol. 61: 1673–1676. 10.1128/aem.61.4.1673-1676.1995 PMC1388429 16535011

[48] Angel R, Matthies D, Conrad R (2011) Activation of methanogenesis in arid biological soil crusts despite the presence of oxygen. Plos One 6: e20453. 21655270 10.1371/journal.pone.0020453 PMC3105065

[49] Ferry JG (1992) Biochemistry of methanogenesis. Crit. Rev. Biochem. Mol. 27: 473–503. 10.3109/10409239209082570 1473352

[50] Bignell DE (1984) Direct potentiometric determination of redox potentials of the gut contents in the termites *Zootermopsis-nevadensis* and *Cubitermes-severus* and in three other arthropods. J. Insect Physiol. 30: 169–174.

[51] Mikhajlova EV (2004) The millipedes (Diplopoda) of the Asian part of Russia. Golovatch Pensoft Series Faunistica I. Sofia, Moscow: Pensoft Publishers. 292 p.

[52] Stoev P, Enghoff H (2008) A revision of the millipede tribe *Apfelbeckiini* Verhoeff, 1900 (Diplopoda: Callipodida: Schizopetalidae). Steenstrupia 30: 47–66.

[53] Hoffman R, Payne J (1969) Diplopods as carnivores. Ecology 50: 1096–1098.

[54] Enghoff H (1992) The size of a millipede. 8th International Congress of Myriapodology, Innsbruck, Austria. Berichte des naturwissenschaftlichen-medizinischen Verein Innsbruck 10: 47–56.

[55] Morozova D, Möhlmann D, Wagner D (2007) Survival of methanogenic Archaea from Siberian permafrost under simulated Martian thermal conditions. Origins Life Evol. B. 37: 189–200. 10.1007/s11084-006-9024-7 17160628

[56] Embley TM, Martin W (1998) Molecular evolution - a hydrogen-producing mitochondrion. Nature 396: 517–519. 9859981 10.1038/24994

[57] Hackstein JHP (2010) Anaerobic ciliates and their methanogenic endosymbionts. In: Hackstein JHP, editor. (Endo)symbiotic methanogenic Archaea. Microbiology Monographs 19. Berlin, Heidelberg: Springer Verlag. 13–23.

[58] Sarmiento BF, Leigh JA, Whitrnan WB (2011) Genetic systems for hydrogenotrophic methanogens. In: Rosenzweig AC, Ragsdale, SW, editors. Methods in Enzymology 494. San Diego: Elsevier Academic Press Inc. 43–73. 10.1016/B978-0-12-385112-3.00003-2 21402209

[59] Brune A (2010) Methanogens in the digestive tracts of termites. In: Hackstein JHP, editor. (Endo)symbiotic Methanogenic Archaea. Microbiology Monographs 19. Berlin, Heidelberg: Springer Verlag. 13–23.

[60] Tokura M, Ohkuma M, Kudo T (2000) Molecular phylogeny of methanogens associated with flagellated protists in the gut and with the gut epithelium of termites. FEMS Microbiol Ecol 33: 233–240. 11098074 10.1111/j.1574-6941.2000.tb00745.x

[61] Zinder SH, Sowers KR, Ferry JG (1985) Notes: *Methanosarcina thermophila* sp. nov., a thermophilic, acetotrophic, methane-producing bacterium. Int. J. Syst. Bacteriol. 35: 522–523.

