# Supplementary material for: Methane Production and Methanogenic Archaea in the Digestive Tracts of Millipedes (Diplopoda)
Source: PLoS One. 2014 Jul 16;9(7):e102659. doi: 10.1371/journal.pone.0102659 (PMC4100924; doi:10.1371/journal.pone.0102659)
Supplement: Table S1 — PCR protocols. Primers and PCR conditions used for amplification of the mcrA gene and the 16S rRNA gene for DGGE analysis. (PDF) [file pone.0102659.s001.pdf]

Table S1. PCR protocols.

Gene	Primers	Primer sequences 5'-3'	PCR cycles	Amplicon length (bp)	Reference for primers
**mcrA -1**	mcrA-F<br>mcrA-R	GGTGGTGTMGGATTCACACARTAYGCWACAGC<br>TTCATTGCRTAGTTWGGRTAGTT	3 min/95 ˚C;<br>30 x (1 min/94 ˚C, 1 min/52 ˚C, 1 min/72 ˚C);<br>10 min/72 ˚C	488	[40]
**mcrA - 2**	ME1-F<br>MCR1-R	GCMATGCARATHGGWATGTC<br>ARCCADATYTGRTCRTA	5 min/95 ˚C;<br>35 x (1 min/94 ˚C, 45 s/59 ˚C, 1 min/72 ˚C);<br>10 min/72 ˚C	280	[41]
**16S rRNA Archaea**	Ar109-F<br>Ar915-R	ACKGCTCAGTAACACGT<br>GTGCTCCCCCGCCAATTCCT	2 min/95 ˚C;<br>20 x (1 min/94 ˚C, 1 min/62** ˚C, 1 min/72 ˚C);<br>10 x (1 min/94 ˚C, 1 min/52 ˚C, 1 min/72 ˚C);<br>10 min/72 ˚C	806	[42]
**16S rRNA methanogens**	MG0357F-gc<br>MG0691R	(GC clamp*)CCCTACGGGGCGCAGCAG<br>GGATTACARGATTTCAC	3 min/94 ˚C;<br>35 x (30 s/94 ˚C, 30 s/53 ˚C, 1 min 30 s/72 ˚C);<br>5 min/72 ˚C	334	[43]

*GC clamp (CGCCCGCCGCGCGCGGCGGGCGGGGCGGGGGCACGGGGGG)
** Touchdown cycling with decrease of 0.5 ˚C in each cycle
