# Supplementary material for: Methane Production and Methanogenic Archaea in the Digestive Tracts of Millipedes (Diplopoda)
Source: PLoS One. 2014 Jul 16;9(7):e102659. doi: 10.1371/journal.pone.0102659 (PMC4100924; doi:10.1371/journal.pone.0102659)
Supplement: Table S2 — Methane production in millipedes and methanogenic microorganisms detected in millipede gut contents and faecal pellets. Methane production was measured by gas chromatography (GC) in the current study or in previous studies, as indicated. The mcrA gene was used as a marker for methanogens in the gut contents and faecal pellets. The presence of methanogens in the gut contents and faecal pellets was also determined by DGGE of the methanogen 16S rRNA gene. Mean values for richness of DGGE profiles are given. (PDF) [file pone.0102659.s002.pdf]

Table S2. Methane production in millipedes and methanogenic microorganisms detected in millipede gut contents and faecal pellets.

Species	Abbr.	Locality	CH_4 prod.	Source of the GC data for CH_4 production	Gut *mcrA-1*	Gut *mcrA-2*	Gut DGGE profile	CH_4 prod. excr.	Excr. *mcrA-1*	Excr. *mcrA-2*	Excr. DGGE profile
**Glomerida**											
Trachysphaeridae											
Trachysphaera costata	**Tco**	SK	**NP**	This study	nd	nd	nd	nd	nd	nd	nd
Glomeridae											
Glomeris hexasticha	**Ghe**	BL , HT	**NP**	This study	-	-	nd	+	+	+	13
Glomeris tetrasticha	**Gte**	SK, HT	**AP**	This study	-	-	8	+	-	-	6
Glomeris balcanica	**Gba**	TH	**NP**	This study	nd	nd	nd	nd	-	-	14
Glomeris marginata	**Gma**	MA	**NP**	This study	nd	nd	nd	nd	nd	nd	nd
Glomeris connexa	**Gco**	MK	**NP**	[23], this study	nd	nd	nd	nd	-	-	22
**Polyzoniida**											
Polyzoniidae											
Polyzonium germanicum	**Pge**	LT	**NP**	This study	nd	nd	nd	nd	nd	nd	nd
Polyzonium eburneum	**Peb**	HT	**NP**	This study	nd	nd	nd	nd	nd	nd	nd
**Chordeumatida**											
Brachychaeteumatidae											
Brachychaeteuma bradeae	**Bbr**	SK	**NP**	This study	nd	nd	nd	nd	nd	nd	nd
Chordeumatidae											
Mycogona germanica	**Mge**	BL	**NP**	This study	nd	nd	nd	nd	nd	nd	nd
Craspedosomatidae											
Craspedosoma transsylvanicum	**Ctr**	ZL	**NP***	[23]	nd	nd	nd	nd	nd	nd	nd
Ochogona caroli	**Oca**	BL	**NP**	This study	nd	nd	nd	nd	nd	nd	nd
Mastigophorophyllidae											
Mastigona mutabilis	**Mmu**	BL, CH	**NP**	This study	nd	nd	nd	nd	nd	nd	nd

Species	Abbr.	Locality	CH_4 prod.	Source of the GC data for CH_4 production	Gut *mcrA-1*	Gut *mcrA-2*	Gut DGGE profile	CH_4 prod. excr.	Excr. *mcrA-1*	Excr. *mcrA-2*	Excr. DGGE profile
**Julida**											
Blaniulidae											
Blaniulus guttulatus	**Bgu**	CB	**NP**	This study	nd	nd	nd	nd	nd	nd	nd
Nopoiulus kochi	**Nko**	PA	**NP**	This study	nd	nd	nd	nd	nd	nd	nd
Julidae											
Cylindroiulus caeruleocinctus	**Cca**	PA	**AP**	This study	-	-	5	+	-	-	19
Megaphyllum unilineatum	**Mun**	TH	**OP**	This study	-	-	7	nd	-	-	12
Leptoiulus trilobatus	**Ltr**	HT, SK	**AP**	This study	-	-	nd	nd	-	-	10
Cylindroiulus luridus	**Clu**	BL	**FP**	This study	-	-	4	++	+	-	8
Julus terrestris	**Jte**	LT	**FP***	[23]	-	-	5	nd	nd	nd	nd
Julus scandinavius	**Jsc**	BL, CH, LT, SK	**FP**	This study	+	-	3	+	-	-	4
Ommatoiulus sabulosus	**Osa**	BK	**OP**	This study	-	-	nd	nd	-	-	nd
Unciger transsilvanicus	**Utr**	BL	**FP***	[23]	nd	nd	nd	nd	nd	nd	nd
Unciger foetidus	**Ufo**	CV, BL, ZL	**AP**	[23], This study	-	-	12	++	+	+	17
Leptoiulus proximus	**Lpr**	LT, SK	**NP**	[23], This study	nd	nd	nd	+	nd	nd	nd
Leptoiulus noricus	**Lno**	BL, CH	**NP**	This study	nd	nd	nd	+	nd	nd	nd
Megaphyllum projectum	**Mpr**	BL, ZL, MK, SK	**AP**	[23], This study	-	-	18	+	-	-	16
Enantiulus nanus	**Ena**	BK	**AP**	This study	nd	nd	nd	++	nd	nd	nd
Tachypodoiulus niger	**Tni**	ST	**NP***	[19]	nd	nd	nd	nd	nd	nd	nd
Cylindroiulus boleti	**Cbo**	LA	**NP***	[23]	nd	nd	nd	nd	nd	nd	nd
Julus curvicornis	**Jcu**	SK	**NP**	This study	nd	nd	nd	+	nd	nd	nd
Megaphyllum sp.	**Me**	ME	**OP**	This study	nd	nd	nd	nd	nd	nd	nd
Pachyiulus hungaricus	**Pah**	ME	**OP**	This study	nd	nd	nd	nd	nd	nd	nd
**Spirobolida**											
Spirobolidae											
***Chicobolus* sp.**	**Chi**	DU	**OP***	[19]	nd	nd	nd	nd	nd	nd	nd
Pachybolidae											
Epibolus pulchripes	**Epu**	MI	**OP**	this study	nd	+	10	+	nd	+	17

Species	Abbr.	Locality	CH_4 prod.	Source of the GC data for CH_4 production	Gut *mcrA-1*	Gut *mcrA-2*	Gut DGGE profile	CH_4 prod. excr.	Excr. *mcrA-1*	Excr. *mcrA-2*	Excr. DGGE profile
**Spirostreptida**											
Spirostreptidae											
***Orthoporus* sp.**	**Or**	ZM	**OP***	[19]	nd	nd	nd	nd	nd	nd	nd
Rhapidostreptus virgator	**Rvi**	ZG	**OP***	[19]	nd	nd	nd	nd	nd	nd	nd
Archispirostreptus gigas	**Agi**	HS	**OP**	This study	+	nd	15	+++	+	+	18
**Callipodida**											
Callipodidae											
Callipodella fasciata	**Cfa**	ME	**NP**	This study	nd	nd	nd	nd	nd	nd	nd
**Polydesmida**											
Aphelidesmidae											
Pycnotropis acuticollis	**Pac**	ZM	**OP***	[19]	nd	nd	nd	nd	nd	nd	nd
Paradoxosomatidae											
Strongylosoma stigmatosum	**Sst**	BL, SK	**AP**	This study	nd	nd	nd	nd	-	-	7
Mestosoma hylaeicum	**Mhy**	ZM	**NP***	[19]	nd	nd	nd	nd	nd	nd	nd
Orthomorpha coarctata	**Oco**	MA	**OP***	This study	nd	nd	nd	nd	nd	nd	nd
Polydesmidae											
Polydesmus complanatus	**Pco**	BL, SK, LT	**NP**	[22], This study	-	-	nd	+	-	-	7
Polydesmus denticulatus	**Pde**	BL	**NP***	[23]	nd	nd	nd	nd	nd	nd	nd
Brachydesmus superus	**Bsu**	PA	**NP**	This study	nd	nd	nd	nd	nd	nd	nd
Trichopolydesmidae											
**not determined**	**T**	SK	**NP**	This study	nd	nd	nd	nd	nd	nd	nd

Abbr. = species abbreviations. **CH_4 prod**. = category after methane production (see text for explanation). **Locality** = localities of collection or breeds: BK – Bohemian Karst Protected Landscape Area (PLA) (Czech Republic), BL – Blanský les PLA (Czech Republic), CB – České Budějovice Basin (Czech Republic), CH – Chelčice (Czech Republic), CV – Českomoravská vrchovina Highlands (Czech Republic), DU – Löbbecke Museum Düsseldorf (Germany), HS – Millipedes obtained from pet shop (M. Kroček, Horní Suchá, Czech Republic). The area of the natural species distribution is Tanzania), HT – Tatra Mts. (Slovakia), LA – Lanžhot (Czech Republic), LT – Latorica PLA (Slovakia), MA – millipedes obtained from breeding in MPI for Terrestrial Microbiology Marburg (Germany). Natural species distribution - Germany (*G. marginata*) and Southeast Asia (*O. coarctata*), ME – Mehedinți Mts. (Romania), MI – millipedes obtained from breeding in Mikulov grammar school (Czech Republic). Natural species distribution - Tanzania, MK – Moravian Karst PLA (Czech Republic), PA – Pálava PLA (Czech Republic), SK – Slovak Karst (Slovakia), ST – Stuttgart (Germany), TH – Thessaloniki (Greece), ZG – Gissen (Germany), ZL – Ždánický les Highlands (Czech Republic), ZM – Marburg (Germany). ***mcrA-1*** and ***mcrA-2*** = detection of *mcrA* genes. **DGGE profile** = numbers of bands obtained from all DGGE profiles (obtained after PCR amplification of the 16S rRNA gene). **gut** = gut content samples. **excr**. = faecal pellets samples. *** -** literature data. **CH_4 prod. excr.** = methane production from faecal pellets, **nd** = not determined, **-** = not detected, and **+** = detected**;** +, ++, and +++ indicate methane production rates ($nl.g^{-1}.h^{-1}$) from faecal pellets of <500, from 500 to 5,000, and > 50.000, respectively.
