# Supplementary material for: Methane Production and Methanogenic Archaea in the Digestive Tracts of Millipedes (Diplopoda)
Source: PLoS One. 2014 Jul 16;9(7):e102659. doi: 10.1371/journal.pone.0102659 (PMC4100924; doi:10.1371/journal.pone.0102659)
Supplement: Table S3 — The sequenced bands of methanogenic archaeal genes amplified from millipedes. Phylogenetic relationships of the excised and sequenced bands of methanogenic archaeal 16S rRNA genes amplified from millipede faecal pellets or gut contents. Sequences that were not submitted to GenBank were those shorter than 200 bp. (PDF) [file pone.0102659.s003.pdf]

Table S3. The sequenced bands of methanogenic archaeal genes amplified from millipedes.

Organism	Isolation source	Band no. (accession no. of sequence)	Methanogenic sequences detected (sequence match)	Accession no. of sequence match	IS	Taxonomy	Pubmed ID
**JULIDAE**							
Julus	faecal pellets	Z1 (KF574068)	*Methanosarcina thermophila* strain KA1	KC203046	99%	Methanomicrobia; Methanosarcinales; Methanosarcinaceae	-
Leptoiulus trilobatus	faecal pellets	Z20 (KF574078)	uncultured *Methanosarcina* sp. clone X4Ar46	AY607271	99%	Methanomicrobia; Methanosarcinales; Methanosarcinaceae	15466514
		Z21 (KF574065)	*Methanosarcina siciliae* type strain DSM3028T	FR733698	99%	Methanomicrobia; Methanosarcinales; Methanosarcinaceae	-
Cylindroiulus caeruleocintus	faecal pellets	Z26 (KF574074)	uncultured Methanomicrobiaceae archaeon clone X4Ar19	AY607244	99%	uncultured Methanomicrobiales / Rice Cluster III archaea	15466514
		Z29 (KF574075)	*Methanosarcina* sp. HC-2	AB288264	99%	Methanomicrobia; Methanosarcinales; Methanosarcinaceae	-
Cylindroiulus caeruleocintus	faecal pellets	ZMG14 (KF574056)	Archaeon LL25A3 16S rRNA gene	AJ745135	99%	Rice Cluster III archaea	15816932
Megaphyllum cf.unilineatum	gut content	ZMG20 (KF574058)	*Methanosarcina* sp. HC-2	AB288264	100%	Methanomicrobia; Methanosarcinales; Methanosarcinaceae	-
Megaphyllum projectum	faecal pellets	Z18 (KF574071)	anaerobic methanogenic archaeon ET1-9	AJ244285	99%	uncultured Methanomicrobiales	10568840
Unciger foetidus	faecal pellets	Z11 (KF574077)	uncultured Methanomicrobiaceae archaeon clone X4Ar19	AY607244	100%	Rice Cluster III archaea	15466514
		Z12 (KF574064)	Candidatus *Methanoregula boonei* strain SN19	EU887826	98%	Methanomicrobia; Methanomicrobiales; Methanoregulaceae	-
Unciger foetidus	faecal pellets	ZMG2 (KF574049)	anaerobic methanogenic archaeon ET1-9	AJ244285	99%	uncultured Methanomicrobiales	10568840
		ZMG3 (KF574050)	anaerobic methanogenic archaeon ET1-9	AJ244285	99%	uncultured Methanomicrobiales	10568840

Organism	Isolation source	Band no. (accession no. of sequence)	Methanogenic sequences detected (sequence match)	Accession no. of sequence match	IS	Taxonomy	Pubmed ID
**GLOMERIDAE**							
Glomeris tetrasticha	gut content	Z23 (KF574073)	uncultured Methanomicrobiales	JF789589	99%	uncultured Methanomicrobiales	21824242
Glomeris tetrasticha	faecal pellets	ZMG1 (KF574048)	uncultured *Methanobrevibacter* sp. clone QTPYAK9	JF807185	100%	Methanobacteria; Methanobacteriales; Methanobacteriaceae	23078429
Glomeris hexasticha	faecal pellets	Z6, Z30 (KF574069, (KF574072)	uncultured Methanomicrobiaceae archaeon clone LrhA51	AJ879031	99%	uncultured Methanomicrobiales	16099988
		Z7 (KF574060)	uncultured Methanosarcinaceae archaeon	AM778298	99%	Methanomicrobia; Methanosarcinales; Methanosarcinaceae	18344350
Glomeris connexa	faecal pellets	Z8-1 (KF574061)	uncultured archaeon isolate DGGE gel band N4	JN546105	99%	Uncultured Methanosarcinales	[39]
		Z8-2 (KF574062)	uncultured soil archaeon partial 16S rRNA gene, isolate 2312F53a	FR745153	99%	Uncultured Methanosarcinales	21478308
		Z9-1 (KF574070)	uncultured Methanomicrobiaceae archaeon clone X4Ar19	AY607244	99%	uncultured Methanomicrobiales	15466514
		Z9-2 (KF574063)	uncultured *Methanosarcina* sp. clone X4Ar35	AY607260	99%	Methanomicrobia; Methanosarcinales; Methanosarcinaceae	15466514
Glomeris balcanica	faecal pellets	Z13 (KF574067)	*Methanobacterium* sp. 17A1	HQ110085	99%	Methanobacteria; Methanobacteriales; Methanobacteriaceae	21890730

Organism	Isolation source	Band no. (accession no. of sequence)	Methanogenic sequences detected (sequence match)	Accession no. of sequence match	IS	Taxonomy	Pubmed ID
**APHELIDESMIDAE**							
Strongylosoma stigmatosum	faecal pellets	Z4 (KF574076)	*Methanobrevibacter woesei* strain CH138	DQ445716	99%	Methanobacteria; Methanobacteriales; Methanobacteriaceae	17085694
		Z3 (KF574059)	*Methanobrevibacter thaueri* strain CW	NR_044787	100%	Methanobacteria; Methanobacteriales; Methanobacteriaceae	-
**SPIROSTREPTIDAE**							
Archispirostreptus gigas	gut content	Z31 (KF574066)	*Methanobrevibacter* sp. gene for 16S rRNA,	AB009827	99%	Methanobacteria; Methanobacteriales; Methanobacteriaceae	10077839
	gut content AHG	ZMG8 (KF574052)	uncultured *Methanobrevibacter* sp. clone RcM20	FJ842696	99%	Methanobacteria; Methanobacteriales; Methanobacteriaceae	-
		ZMG10 (KF574053)	*Methanobrevibacter arboriphilus* strain ANOR1	KC616344	98%	Methanobacteria; Methanobacteriales; Methanobacteriaceae	-
	gut content PHG	ZMG11 (KF574054)	uncultured euryarchaeote clone P4b-Ar-8	AF293505	98%	uncultured Methanobacteriales	11571197
		ZMG12 (KF574055)	uncultured archaeon clone PeHAr11	AJ576151	99%	uncultured Methanobacteriales	14602626
Archispirostreptus gigas	faecal pellets	ZMG6 (KF574051)	uncultured archaeon clone ARD1A50TFC	JX906097	98%	uncultured Methanobacteriales	23144861
		Z37 (not submitted)	Methanogenic archaeon LGM-AFM02	EU863826	98%	uncultured Methanobacteriales	21719276

Organism	Isolation source	Band no. (accession no. of sequence)	Methanogenic sequences detected (sequence match)	Accession no. of sequence match	IS	Taxonomy	Pubmed ID
**PACHYBOLIDAE**							
Epibous pulchripes	faecal pellets	S16 (KF739300)	uncultured euryarchaeote clone P3-Ar-28	AF293573	98%	Methanobacteria; Methanobacteriales; Methanobacteriaceae	11571197
		S17 (KF739301)	uncultured *Methanobrevibacter* sp. clone RcM20	FJ842696	99%	Methanobacteria; Methanobacteriales; Methanobacteriaceae	-
	gut content	S19 (KF739303)	uncultured archaeon clone: NtAr-G95	AB284305	99%	Methanobacteria; Methanobacteriales; Methanobacteriaceae	-
		S20 (KF739304)	uncultured *Methanobrevibacter* sp. clone RcM20	FJ842696	99%	Methanobacteria; Methanobacteriales; Methanobacteriaceae	-

AHG = anterior part of hingut, **PHG** = posterior part of hindgut
